# The genome sequence of the Shuttle-shaped Dart, *Agrotis puta *(Hübner, 1803)

**DOI:** 10.12688/wellcomeopenres.18934.1

**Published:** 2023-02-15

**Authors:** Douglas Boyes, Gavin R. Broad, University of Oxford and Wytham Woods Genome Acquisition Lab, Natural History Museum Genome Acquisition Lab, Darwin Tree of Life Barcoding collective, Wellcome Sanger Institute Tree of Life programme, Wellcome Sanger Institute Scientific Operations: DNA Pipelines collective, Tree of Life Core Informatics collective, Peter W.H. Holland, Darwin Tree of Life Consortium

**Affiliations:** 1 UK Centre for Ecology and Hydrology, Wallingford, Oxfordshire, UK; 2 Natural History Museum, London, UK; 3 University of Oxford, Oxford, Oxfordshire, UK

**Keywords:** Agrotis puta, Shuttle-shaped Dart, genome sequence, chromosomal, Lepidoptera

## Abstract

We present a genome assembly from an individual male *Agrotis puta* (the Shuttle-shaped Dart; Arthropoda; Insecta; Lepidoptera; Noctuidae). The genome sequence is 522 megabases in span. Most of the assembly is scaffolded into 31 chromosomal pseudomolecules, including the assembled Z chromosome. The mitochondrial genome has also been assembled and is 15.4 kilobases in length. Gene annotation of this assembly on Ensembl has identified 15,136 protein coding genes.

## Species taxonomy

Eukaryota; Metazoa; Ecdysozoa; Arthropoda; Hexapoda; Insecta; Pterygota; Neoptera; Endopterygota; Lepidoptera; Glossata; Ditrysia; Noctuoidea; Noctuidae; Noctuinae; Noctuini; *Agrotis*; *Agrotis puta* (Hübner, 1803) (NCBI:txid1857961)

## Background

The Shuttle-shaped Dart *Agrotis puta* is a moth in the family Noctuidae found across most of Europe, with its range extending into parts of Scandinavia and North Africa ( GBIF Secretariat, 2022). In the UK, the adult moth is common across the south of England and Wales, and it has also been recorded in smaller numbers in northern England and Scotland ( NBN Atlas, 2021; Randle *et al*., 2019). There are very few records from Ireland with the first record in 1984 ( Randle *et al.*, 2019). The adult is on the wing from May to September in the UK, with occasional records earlier and later in the year. Larvae feed on a range of herbaceous plants, including dandelion and dock. There are two or three generations per year in southern England with the last generation overwintering as a larva ( Randle *et al.*, 2019; South, 1961) 

The common name of the moth derives from a pointed lozenge-shaped mark, outlined in cream, on the forewing: the shape resembles the wooden ‘shuttle’ used by weavers to carry a spool of wool or other thread during weaving of cloth. This diagnostic wing marking stands out from the otherwise relatively plain wing, usually dark brown in females and pale yellowish brown in males. Bilateral gynandromorphs have been reported, with the wing patterning being male on one side of the midline and female on the other ( South, 1961; UKMoths, no date).

A genome sequence for *Agrotis put*a will facilitate research into the genetic basis of sexual dimorphism in colour pattern, and contribute to the growing resource for comparative genomic studies across the Lepidoptera.

## Genome sequence report

**Figure 1.**
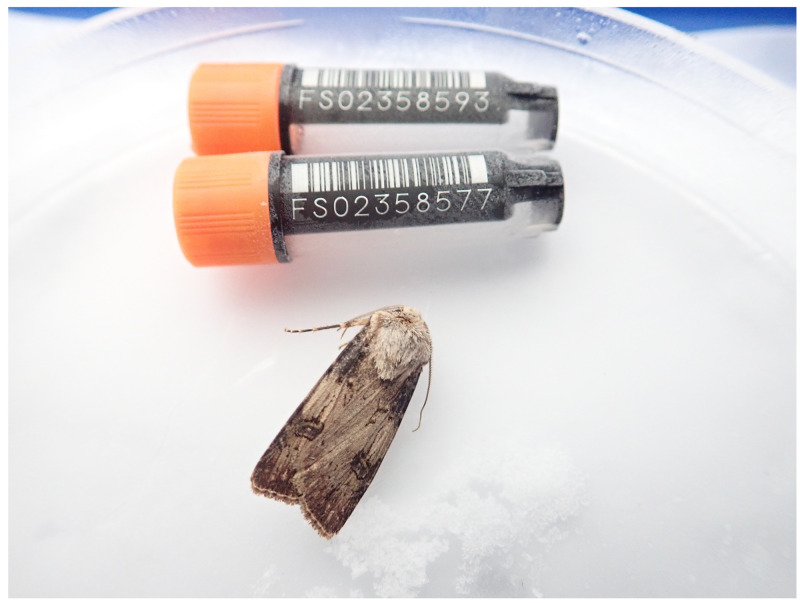
Photograph of the *Agrotis puta* (ilAgrPuta1) specimen used for genome sequencing.

The genome was sequenced from an individual *A. puta* specimen ( Figure 1) collected in Wytham Woods, UK (latitude 51.77, longitude –1.34). A total of 47-fold coverage in Pacific Biosciences single-molecule HiFi long reads and 89-fold coverage in 10X Genomics read clouds were generated. Primary assembly contigs were scaffolded with chromosome conformation Hi-C data. Manual assembly curation corrected 27 missing joins or mis-joins and removed three haplotypic duplications, reducing the scaffold number by 2.78%, and decreasing the scaffold N50 by 4.16%.

**Figure 2.**
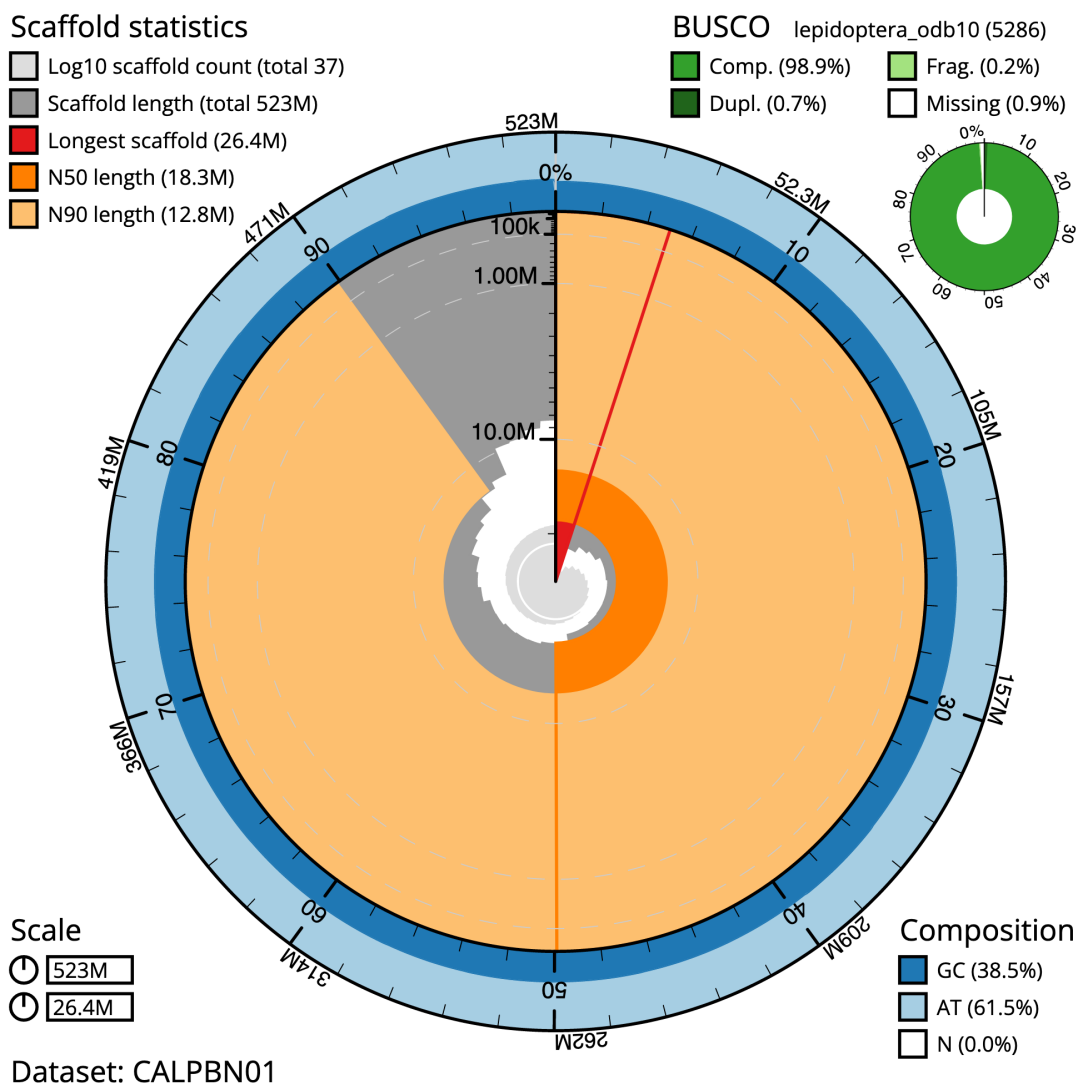
Genome assembly of *Agrotis puta*, ilAgrPuta1.2: metrics. The BlobToolKit Snailplot shows N50 metrics and BUSCO gene completeness. The main plot is divided into 1,000 size-ordered bins around the circumference with each bin representing 0.1% of the 523,321,491 bp assembly. The distribution of scaffold lengths is shown in dark grey with the plot radius scaled to the longest scaffold present in the assembly (26,353,403 bp, shown in red). Orange and pale-orange arcs show the N50 and N90 scaffold lengths (18,289,922 and 12,814,946 bp), respectively. The pale grey spiral shows the cumulative scaffold count on a log scale with white scale lines showing successive orders of magnitude. The blue and pale-blue area around the outside of the plot shows the distribution of GC, AT and N percentages in the same bins as the inner plot. A summary of complete, fragmented, duplicated and missing BUSCO genes in the lepidoptera_odb10 set is shown in the top right. An interactive version of this figure is available at https://blobtoolkit.genomehubs.org/view/ilAgrPuta1.1/dataset/CALPBN01/snail.

The final assembly has a total length of 522.1 Mb in 35 sequence scaffolds with a scaffold N50 of 18.2 Mb ( Table 1). Most (99.98%) of the assembly sequence was assigned to 31 chromosomal-level scaffolds, representing 30 autosomes and the Z sex chromosome. Chromosome-scale scaffolds confirmed by the Hi-C data are named in order of size ( Figure 2– Figure 5; Table 2). The assembly has a BUSCO v5.3.2 ( Manni *et al.*, 2021) completeness of 98.9% (single 98.2%, duplicated 0.7%) using the lepidoptera_odb10 reference set. While not fully phased, the assembly deposited is of one haplotype. Contigs corresponding to the second haplotype have also been deposited.

**Table 1.** Genome data for *Agrotis puta*, ilAgrPuta1.2.

Project accession data		
Assembly identifier	ilAgrPuta1.2	
Species	*Agrotis puta*	
Specimen	ilAgrPuta1; ilAgrPuta2	
NCBI taxonomy ID	1857961	
BioProject	PRJEB52475	
BioSample ID	SAMEA7701549	
Isolate information	ilAgrPuta1; abdomen (PacBio, 10X), head and thorax (Hi-C) ilAgrPuta2: thorax (RNA-Seq)	
Assembly metrics *		*Benchmark*
Consensus quality (QV)	60.8	*≥ 50*
*k*-mer completeness	100%	*≥ 95%*
BUSCO **	C:98.9%[S:98.2%,D:0.7%], F:0.2%,M:0.9%,n:5,286	*C ≥ 95%*
Percentage of assembly mapped to chromosomes	99.98%	*≥ 95%*
Sex chromosomes	Z chromosome	*localised homologous pairs*
Organelles	Mitochondrial genome assembled	*complete single alleles*
Raw data accessions		
PacificBiosciences SEQUEL II	ERR9682743, ERR9682744	
10X Genomics Illumina	ERR9682478–ERR9682481	
Hi-C Illumina	ERR9682477	
PolyA RNA-Seq Illumina	ERR10123694	
Genome assembly		
Assembly accession	GCA_943136025.2	
*Accession of alternate haplotype*	GCA_943137145.2	
Span (Mb)	522.1	
Number of contigs	72	
Contig N50 length (Mb)	12.0	
Number of scaffolds	35	
Scaffold N50 length (Mb)	18.2	
Longest scaffold (Mb)	26.6	
Genome annotation		
Number of protein-coding genes	15,136	
Number of non-coding genes	4,529	
Number of gene transcripts	29,573	

* Assembly metric benchmarks are adapted from column VGP-2020 of “Table 1: Proposed standards and metrics for defining genome assembly quality” from ( Rhie *et al.*, 2021). ** BUSCO scores based on the lepidoptera_odb10 BUSCO set using v5.3.2 C = complete [S = single copy, D = duplicated], F = fragmented, M = missing, n = number of orthologues in comparison. A full set of BUSCO scores is available at https://blobtoolkit.genomehubs.org/view/ilAgrPuta1.1/dataset/CALPBN01/busco.

**Figure 3.**
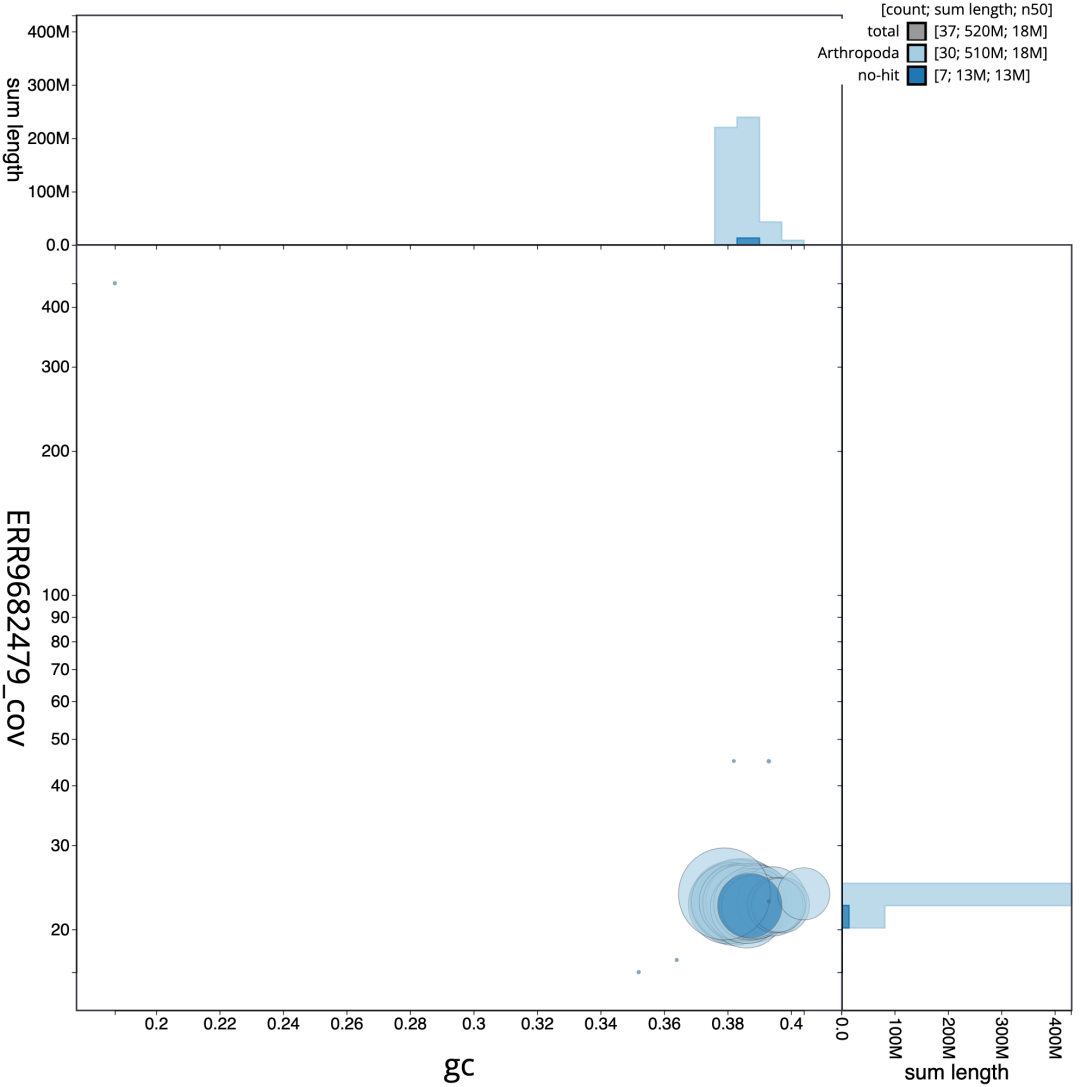
Genome assembly of *Agrotis puta*, ilAgrPuta1.2: GC coverage. BlobToolKit GC-coverage plot. Scaffolds are coloured by phylum. Circles are sized in proportion to scaffold length. Histograms show the distribution of scaffold length sum along each axis. An interactive version of this figure is available at https://blobtoolkit.genomehubs.org/view/ilAgrPuta1.1/dataset/CALPBN01/blob.

**Figure 4.**
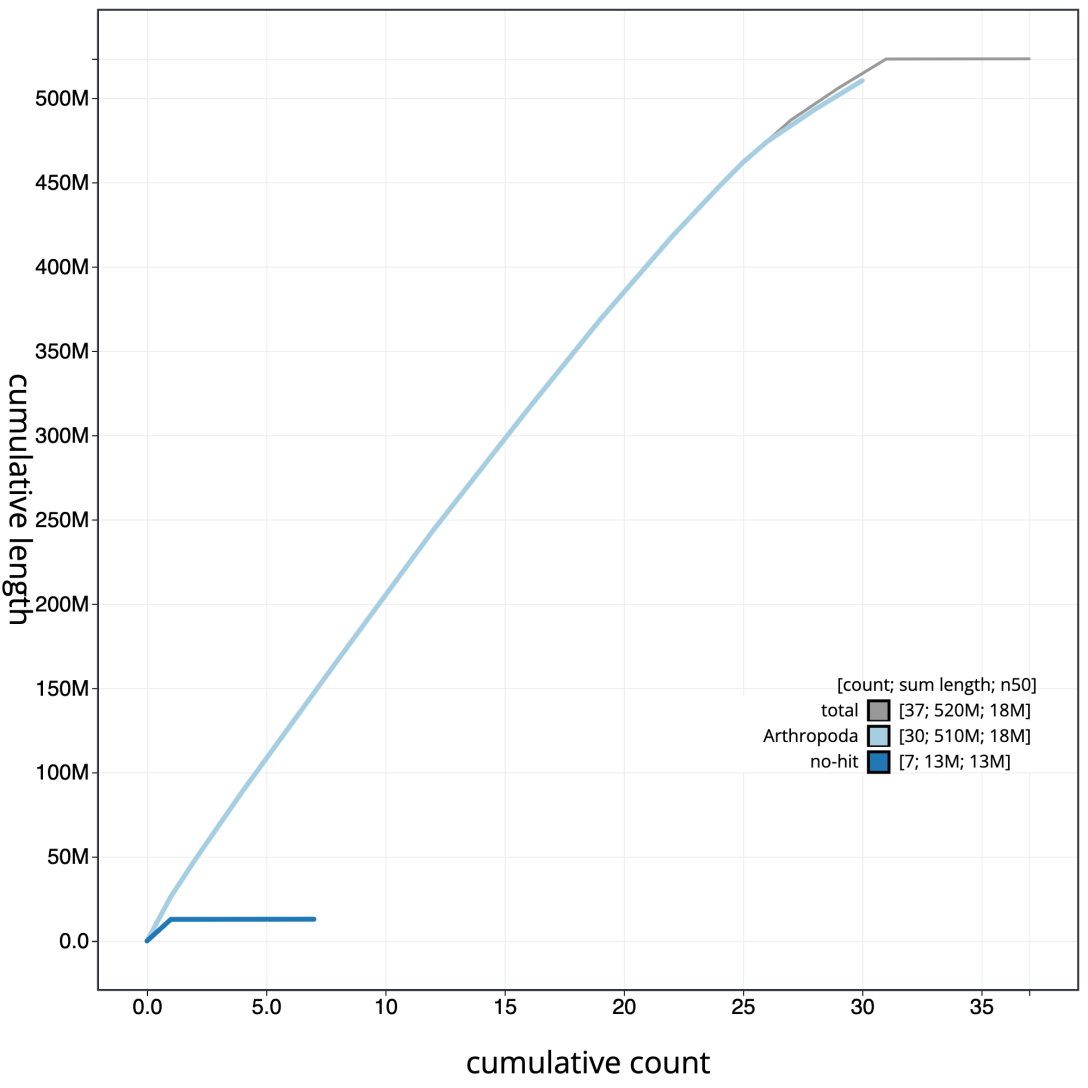
Genome assembly of *Agrotis puta*, ilAgrPuta1.2: cumulative sequence. BlobToolKit cumulative sequence plot. The grey line shows cumulative length for all scaffolds. Coloured lines show cumulative lengths of scaffolds assigned to each phylum using the buscogenes taxrule. An interactive version of this figure is available at https://blobtoolkit.genomehubs.org/view/ilAgrPuta1.1/dataset/CALPBN01/cumulative.

**Figure 5.**
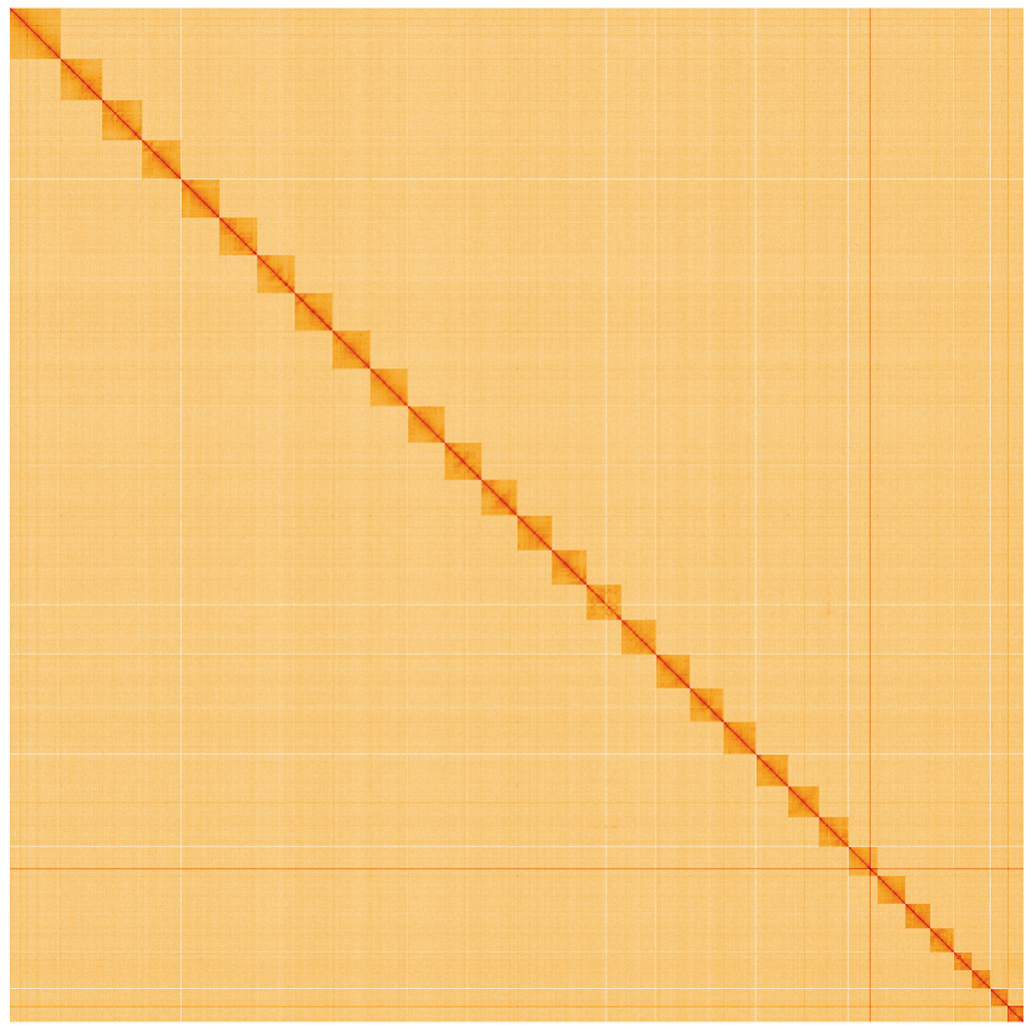
Genome assembly of *Agrotis puta*, ilAgrPuta1.2: Hi-C contact map. Hi-C contact map of the ilAgrPuta1.2 assembly, visualised using HiGlass. Chromosomes are shown in order of size from left to right and top to bottom. An interactive version of this figure may be viewed at https://genome-note-higlass.tol.sanger.ac.uk/l/?d=H_16A46HT2ybsNtwOFdvSQ.

**Table 2.** Chromosomal pseudomolecules in the genome assembly of *Agrotis puta*, ilAgrPuta1.

INSDC accession	Chromosome	Size (Mb)	GC content (%)
OW964167.2	1	21.11	38
OW964168.2	2	20.69	38
OW964169.2	3	20.42	38.5
OW964170.2	4	19.88	38.5
OW964171.2	5	19.65	38
OW964172.2	6	19.71	38
OW964173.2	7	19.29	38
OW964174.2	8	19.47	38
OW964175.2	9	19.29	38.5
OW964176.2	10	19.43	38
OW964177.2	11	18.79	38
OW964178.2	12	18.09	38
OW964179.2	13	18.15	38
OW964180.2	14	17.83	38
OW964181.2	15	17.58	38.5
OW964182.2	16	17.64	38
OW964183.2	17	17.34	38.5
OW964184.2	18	17.40	38.5
OW964185.2	19	16.60	38.5
OW964186.2	20	16.19	38.5
OW964187.2	21	16.13	38.5
OW964188.2	22	15.31	38.5
OW964189.2	23	14.45	39
OW964190.2	24	14.33	38.5
OW964191.2	25	12.60	38.5
OW964192.2	26	12.18	38.5
OW964193.2	27	9.01	39.5
OW964194.2	28	9.48	39
OW964195.2	29	9.01	39.5
OW964196.2	30	8.44	40
OW964166.2	Z	26.55	37.5
OW964197.2	MT	0.02	18.5

## Genome annotation report

The *A. puta* genome assembly GCA_943136025.1 was annotated using the Ensembl rapid annotation pipeline ( Table 1; https://rapid.ensembl.org/Agrotis_puta_GCA_943136025.1/). The resulting annotation includes 29,573 transcribed mRNAs from 15,136 protein-coding and 4,529 non-coding genes.

## Methods

### Sample acquisition and nucleic acid extraction

An individual *A. puta* specimen (ilAgrPuta1) was collected in Wytham Woods, Oxfordshire (biological vice-county: Berkshire), UK (latitude 51.77, longitude –1.34) on 20 July 2020, using a light trap. The specimens were collected and identified by Douglas Boyes (University of Oxford) and snap-frozen on dry ice. This specimen was used for DNA and Hi-C sequencing.

A second *A. puta* specimen (ilAgrPuta2) was caught in Tonbridge, Kent, UK (latitude 51.19, longitude 0.29) on 20 August 2020 by Gavin Broad (Natural History Museum) and preserved on dry ice. This specimen was used for RNA sequencing.

DNA was extracted at the Tree of Life laboratory, Wellcome Sanger Institute (WSI). The ilAgrPuta1 sample was weighed and dissected on dry ice with tissue set aside for Hi-C sequencing. Abdomen tissue was disrupted using a Nippi Powermasher fitted with a BioMasher pestle. High molecular weight (HMW) DNA was extracted using the Qiagen MagAttract HMW DNA extraction kit. Low molecular weight DNA was removed from a 20 ng aliquot of extracted DNA using 0.8X AMpure XP purification kit prior to 10X Chromium sequencing; a minimum of 50 ng DNA was submitted for 10X sequencing. HMW DNA was sheared into an average fragment size of 12–20 kb in a Megaruptor 3 system with speed setting 30. Sheared DNA was purified by solid-phase reversible immobilisation using AMPure PB beads with a 1.8X ratio of beads to sample to remove the shorter fragments and concentrate the DNA sample. The concentration of the sheared and purified DNA was assessed using a Nanodrop spectrophotometer and Qubit Fluorometer and Qubit dsDNA High Sensitivity Assay kit. Fragment size distribution was evaluated by running the sample on the FemtoPulse system.

RNA was extracted from thorax tissue of ilAgrPuta2 in the Tree of Life Laboratory at the WSI using TRIzol, according to the manufacturer’s instructions. RNA was then eluted in 50 μl RNAse-free water and its concentration assessed using a Nanodrop spectrophotometer and Qubit Fluorometer using the Qubit RNA Broad-Range (BR) Assay kit. Analysis of the integrity of the RNA was done using Agilent RNA 6000 Pico Kit and Eukaryotic Total RNA assay.

### Sequencing

Pacific Biosciences HiFi circular consensus and 10X Genomics read cloud DNA sequencing libraries were constructed according to the manufacturers’ instructions. Poly(A) RNA-Seq libraries were constructed using the NEB Ultra II RNA Library Prep kit. DNA and RNA sequencing was performed by the Scientific Operations core at the WSI on Pacific Biosciences SEQUEL II (HiFi) and Illumina NovaSeq 6000 (RNA-Seq and 10X) instruments. Hi-C data were also generated from head and thorax tissue of ilAgrPuta1 using the Arima v2 kit and sequenced on the Illumina NovaSeq 6000 instrument.

### Genome assembly

Assembly was carried out with Hifiasm ( Cheng *et al.*, 2021) and haplotypic duplication was identified and removed with purge_dups ( Guan *et al.*, 2020). One round of polishing was performed by aligning 10X Genomics read data to the assembly with Long Ranger ALIGN, calling variants with freebayes ( Garrison & Marth, 2012). The assembly was then scaffolded with Hi-C data ( Rao *et al.*, 2014) using YaHS ( Zhou *et al.*, 2022). The assembly was checked for contamination and corrected as described previously ( Howe *et al.*, 2021). Manual curation was performed using HiGlass ( Kerpedjiev *et al.*, 2018) and Pretext ( Harry, 2022). The mitochondrial genome was assembled using MitoHiFi ( Uliano-Silva *et al.*, 2022), which performed annotation using MitoFinder ( Allio *et al.*, 2020). The genome was analysed and BUSCO scores were generated within the BlobToolKit environment ( Challis *et al.*, 2020). Table 3 contains a list of all software tool versions used, where appropriate.

**Table 3.** Software tools and versions used.

Software tool	Version	Source
BlobToolKit	3.5.0	Challis *et al.*, 2020
freebayes	1.3.1-17-gaa2ace8	Garrison & Marth, 2012
Hifiasm	0.16.1-r375	Cheng *et al.*, 2021
HiGlass	1.11.6	Kerpedjiev *et al.*, 2018
Long Ranger ALIGN	2.2.2	https:// support.10xgenomics. com/genome-exome/ software/pipelines/ latest/advanced/other- pipelines
MitoHiFi	2	Uliano-Silva *et al.*, 2022
PretextView	0.2	Harry, 2022
purge_dups	1.2.3	Guan *et al.*, 2020
YaHS	yahs-1.1.91eebc2	Zhou *et al.*, 2022

### Genome annotation

The Ensembl gene annotation system ( Aken *et al.*, 2016) was used to generate annotation for the *A. puta* assembly (GCA_943136025.1). Annotation was created primarily through alignment of transcriptomic data to the genome, with gap filling via protein to-genome alignments of a select set of proteins from UniProt ( UniProt Consortium, 2019).

### Ethics/compliance issues

The materials that have contributed to this genome note have been supplied by a Darwin Tree of Life Partner. The submission of materials by a Darwin Tree of Life Partner is subject to the Darwin Tree of Life Project Sampling Code of Practice. By agreeing with and signing up to the Sampling Code of Practice, the Darwin Tree of Life Partner agrees they will meet the legal and ethical requirements and standards set out within this document in respect of all samples acquired for, and supplied to, the Darwin Tree of Life Project. Each transfer of samples is further undertaken according to a Research Collaboration Agreement or Material Transfer Agreement entered into by the Darwin Tree of Life Partner, Genome Research Limited (operating as the Wellcome Sanger Institute), and in some circumstances other Darwin Tree of Life collaborators.

## Data Availability

European Nucleotide Archive: *Agrotis puta* (shuttle-shaped dart). Accession number PRJEB52475; https://identifiers.org/ena.embl/PRJEB52475. ( Wellcome Sanger Institute, 2022) The genome sequence is released openly for reuse. The *Agrotis puta* genome sequencing initiative is part of the Darwin Tree of Life (DToL) project. All raw sequence data and the assembly have been deposited in INSDC databases. Raw data and assembly accession identifiers are reported in Table 1.

## Author information

Members of the University of Oxford and Wytham Woods Genome Acquisition Lab are listed here: https://doi.org/10.5281/zenodo.4789928.

Members of the Natural History Museum Genome Acquisition Lab are listed here: https://doi.org/10.5281/zenodo.4790042.

Members of the Darwin Tree of Life Barcoding collective are listed here: https://doi.org/10.5281/zenodo.4893703.

Members of the Wellcome Sanger Institute Tree of Life programme are listed here: https://doi.org/10.5281/zenodo.4783585.

Members of Wellcome Sanger Institute Scientific Operations: DNA Pipelines collective are listed here: https://doi.org/10.5281/zenodo.4790455.

Members of the Tree of Life Core Informatics collective are listed here: https://doi.org/10.5281/zenodo.5013541.

Members of the Darwin Tree of Life Consortium are listed here: https://doi.org/10.5281/zenodo.4783558.
