# Supplementary material for: The genome sequence of the Shuttle-shaped Dart, Agrotis puta (Hübner, 1803)
Source: Wellcome Open Res. 2023 Feb 15;8:84. [Version 1] doi: 10.12688/wellcomeopenres.18934.1 (PMC10879758; doi:10.12688/wellcomeopenres.18934.1)
Supplement: Peer review history

# Reviewer report: Reviewer response for version 1

**DOI:** 10.21956/wellcomeopenres.20992.r72572

**Published:** 2024-02-20

**Authors:** Qingsong Zhou

**Affiliations:** 1 Chinese Academy of Sciences, Beijing, China

The authors presented the genome of the Shuttle-shaped Dart, *Agrotis puta* at the chromosome level. The methods and software used in the analysis workflow were effectively employed to generate reference genome of Lepidoptera. The BlobToolKit and BUSCO assessments show that the genome is of high quality. It would be better to provide more detailed information for the annotated genes. In conclusion, this work contributes to the reference genome resources of Lepidoptera.

Are sufficient details of methods and materials provided to allow replication by others?

Yes

Is the rationale for creating the dataset(s) clearly described?

Yes

Are the datasets clearly presented in a useable and accessible format?

Yes

Are the protocols appropriate and is the work technically sound?

Yes

Reviewer Expertise:

Entomology, genomics

I confirm that I have read this submission and believe that I have an appropriate level of expertise to confirm that it is of an acceptable scientific standard.

# Reviewer report: Reviewer response for version 1

**DOI:** 10.21956/wellcomeopenres.20992.r72570

**Published:** 2024-02-13

**Authors:** Barbara Feldmeyer

**Affiliations:** 1 Senckenberg Biodiversity and Climate Research Centre, Frankfurt am Main, Germany

This manuscript presents a high-quality reference genome assembly for the Shuttle-shaped Dart, Agrotis puta, from a single male individual. The genome sequence spans 522Mb in length and is anchored into 31 chromosome-scaled scaffolds, including the Z chromosome. Genome annotation identified 15,136 protein-coding genes with 98.9% BUSCO completeness. The mitochondrial genome has also been assembled and annotated. 

 This is a very well-written report, and there are only three aspects I would like the authors to address:

- I trust that "The adult is on the wing from..." is correct? If colloquial please change to "active from May to September".
- Please add information on which tissue was actually used, or was the gut removed and the rest used for DNA extraction,... . There must be a reason why you dissected.
- I couldn't find information on the submission strategy of the mitochondrial genome. Was included in the assembly.fasta, do you have an extra accession number for the mtGenome, and what about the mt-annotation? Please add this information.

Are sufficient details of methods and materials provided to allow replication by others?

Partly

Is the rationale for creating the dataset(s) clearly described?

Yes

Are the datasets clearly presented in a useable and accessible format?

Yes

Are the protocols appropriate and is the work technically sound?

Yes

Reviewer Expertise:

molecular ecology

I confirm that I have read this submission and believe that I have an appropriate level of expertise to confirm that it is of an acceptable scientific standard, however I have significant reservations, as outlined above.

# Reviewer report: Reviewer response for version 1

**DOI:** 10.21956/wellcomeopenres.20992.r59757

**Published:** 2023-07-14

**Authors:** Jason Hill

**Affiliations:** 1 Science for Life Laboratory, Department of Medical Biochemistry and Microbiology, Uppsala University, Uppsala, Sweden

The authors assembled and annotated the genome of of the Shuttle-shaped Dart, *Agrotis puta. *The methods employed represent an excellent application of the best available methods for generating reference sequences of Lepidoptera. While no particular hypotheses were tested here, this work represents a valuable contribution to the resources available for further work.

Are sufficient details of methods and materials provided to allow replication by others?

Yes

Is the rationale for creating the dataset(s) clearly described?

Yes

Are the datasets clearly presented in a useable and accessible format?

Yes

Are the protocols appropriate and is the work technically sound?

Yes

Reviewer Expertise:

Population genomics.

I confirm that I have read this submission and believe that I have an appropriate level of expertise to confirm that it is of an acceptable scientific standard.
